# Remediating Biotoxicity with Synthetic Receptors

**DOI:** 10.1021/acscentsci.6c00706

**Published:** 2026-05-11

**Authors:** Richard J. Hooley

**Affiliations:** Department of Chemistry, University of California − Riverside, Riverside, California 92521, United States

## Abstract

Water-soluble synthetic receptors selectively bind α-synuclein in cells and disrupt the formation of prion-like aggregates, remediating the toxic effects of aggregation.

## Graphical abstract

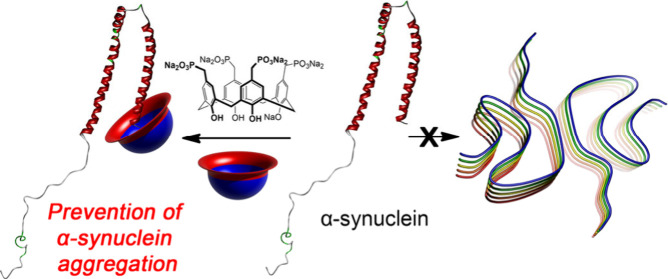

Macrocyclic synthetic receptors, often inspired by natural proteins and enzymes, are frequently touted as “biomimetic” species capable of functions only shown by biomolecules.^1^ This is true in many cases, and spectacular achievements have been shown in biomimetic catalysis,^2^ cargo delivery, and molecular recognition.^3^ The key to all these properties lies in the presence of a defined cavity that allows size- and shape-selective, noncovalent molecular recognition to form receptor:substrate complexes, mirroring the mechanism of Michaelis complex formation between enzymes and their substrates. However, function in complex environments, such as cells, remains a challenge.^4^ The presence of multiple interferents in the medium makes *selective* molecular recognition challenging for simple macrocycles. These interferents can be as simple as inorganic salts—for example, high concentrations of Na^+^ cations can limit the utility of cucurbiturils in aqueous solution.^5^ Alternatively, when trying to coordinate specific residues on a protein, one must consider interference from other molecular species of similar or identical structure—how does one selectively bind a specific lysine residue in the presence of multiple arginines, lysines, and the N-terminus in a protein?

Simply binding to a specific protein in a cellular environment with a synthetic receptor is challenging enough, but changing the cellular properties of that protein adds an extra layer of complexity.

All these challenges must be considered when attempting to perform selective biorecognition in complex media, but they are exacerbated when trying to confer *function* on the process. Simply binding to a specific protein in a cellular environment with a synthetic receptor is challenging enough, but changing the cellular properties of that protein adds an extra layer of complexity. In this issue of *ACS Central Science*, Prof. Francesco Sansone, Prof. Roberta Ruotolo, and co-workers show that water-soluble calixarenes can coordinate α-synuclein proteins in cells, and prevent their aggregation, lowering the toxicity of the protein aggregates.^6^ The authors show that functionalized phosphonate calixarenes can selectively bind to α-synuclein in yeast cells despite their relatively simple structure, and this selective binding is strong enough to disrupt the protein:protein interactions necessary for α-synuclein aggregation. The use of minimally functionalized calixarenes for this process is impressive, and quite surprising. While others have shown that calixarenes can be synthetically tailored for biomedical applications, including drug delivery, phototheranostics, and bioimaging, those calixarenes are specifically designed to self-aggregate into nanoparticle-sized micellar complexes.^7^ The large, complex structure both protects the calixarenes from off-target processes in the cell, and enables selective molecular recognition of suitably sized large targets. In contrast, the work shown here uses relatively simple calixarene scaffolds, with only phosphonate groups at the upper rim for selective protein binding and aggregate disruption in a cell.

The authors tested a series of water-soluble calixarenes, appended with either sulfonyl or phosphonyl groups to confer water-solubility on the receptor and to control protein recognition. Surprisingly, the phosphonylated calixarenes were selective for α-synuclein aggregate disruption, whereas the sulfonated variants showed cytotoxicity. Sulfonyl and phosphonyl groups are commonly thought of as isosteres, as they are very similar in size, shape, hydration shell, and charge, but this work shows that subtle differences in structure can have large effects on biorecognition and efficacy in the complex environment of a cell.

The interactions between the receptor and α-synuclein were studied by ^15^N–^1^H HSQC NMR analysis (Figure 1), which showed perturbations localized at the N-terminal region of the protein. There is a preponderance of lysines at the N-terminus, which is consistent with other studies that show charged calixarenes bind folded proteins at externally oriented cationic sites in the solid state and in solution.^8,9^ The recognition sites are located around the early KTKEGV consensus motifs—these α-helical regions mediate the initial contact points for α-synuclein assembly,^10^ and so the selective binding of the calixarene macrocycle at this position is highly effective at disrupting initial aggregation, preventing prion-like aggregate growth.

**Figure 1:**
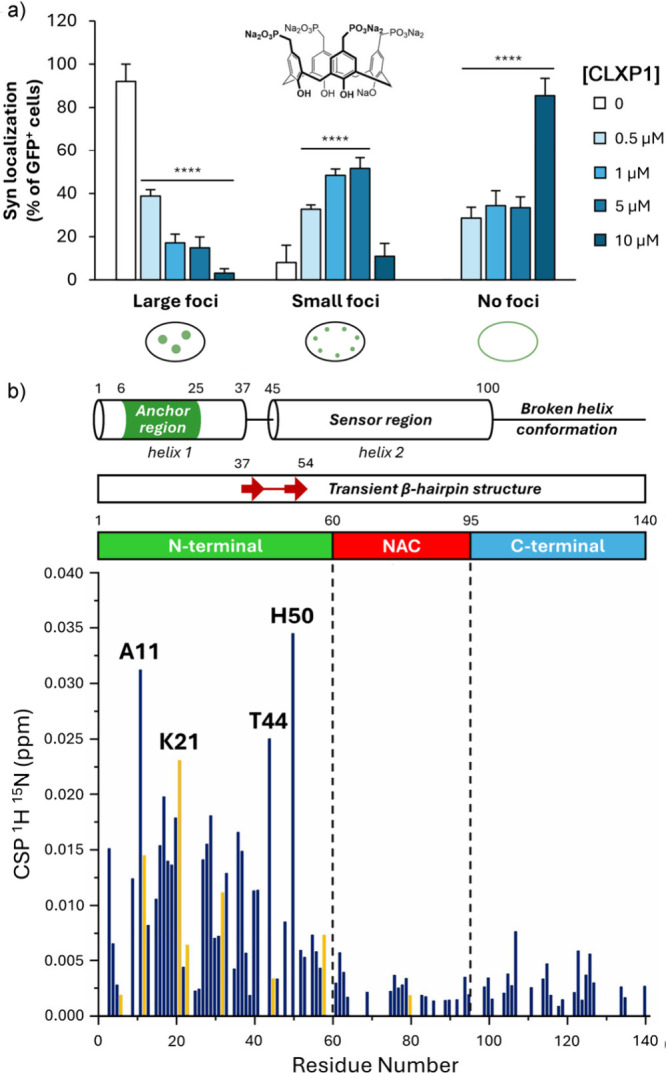
(a) Percentage of GFP+ cells displaying intracellular foci (large or small) or no detectable foci (with syn predominantly localized at the plasma membrane) calculated relative to the total number of GFP+ cells treated with varying concentrations of **CLXP1**. (b) Plot of ^1^H–^15^N chemical shift perturbation (CSP) values versus residue number at 1:9.6 syn:**CLXP1** molar ratio, illustrating the localization of **CLXP1** binding at the α-synuclein N-terminus. Reproduced with permission from ref 6. Available under a CC-BY 4.0 license. Copyright 2026 Davide Dell’Accantera, Giulia Piccinini, Cristina Ciabini, Isabella C. Felli, Stefano Volpi, Nelson Marmiroli, Francesco Sansone, Roberta Ruotolo.

Interestingly, the most effective calixarene tested was **CLXP1** (Figure 2), which contains hydroxyl groups at the lower rim as well as the coordinating upper rim phosphonates. Protection of these hydroxyl groups reduced the cytoprotective effects, and this has been ascribed to antioxidant capabilities of the hydroxylated calixarene. Hydroxycalixarenes are reminiscent of polyphenols in structure, and the authors show that **CLXP1** also reduced oxidative stress in the cell. The combination of both phosphonylation at the upper rim and free hydroxyls at the lower rim provide the best synergy between aggregation disruption and antioxidant properties, showing the greatest cytoprotective effects. The “dual-mode” action of the receptor is essential for efficacy, showing the delicate interplay of properties that can occur when mediating cellular processes, and illustrating the challenge in using receptors as therapeutics—minuscule changes in structure can have very large effects on molecular interactions, including off-target interactions.

**Figure 2:**
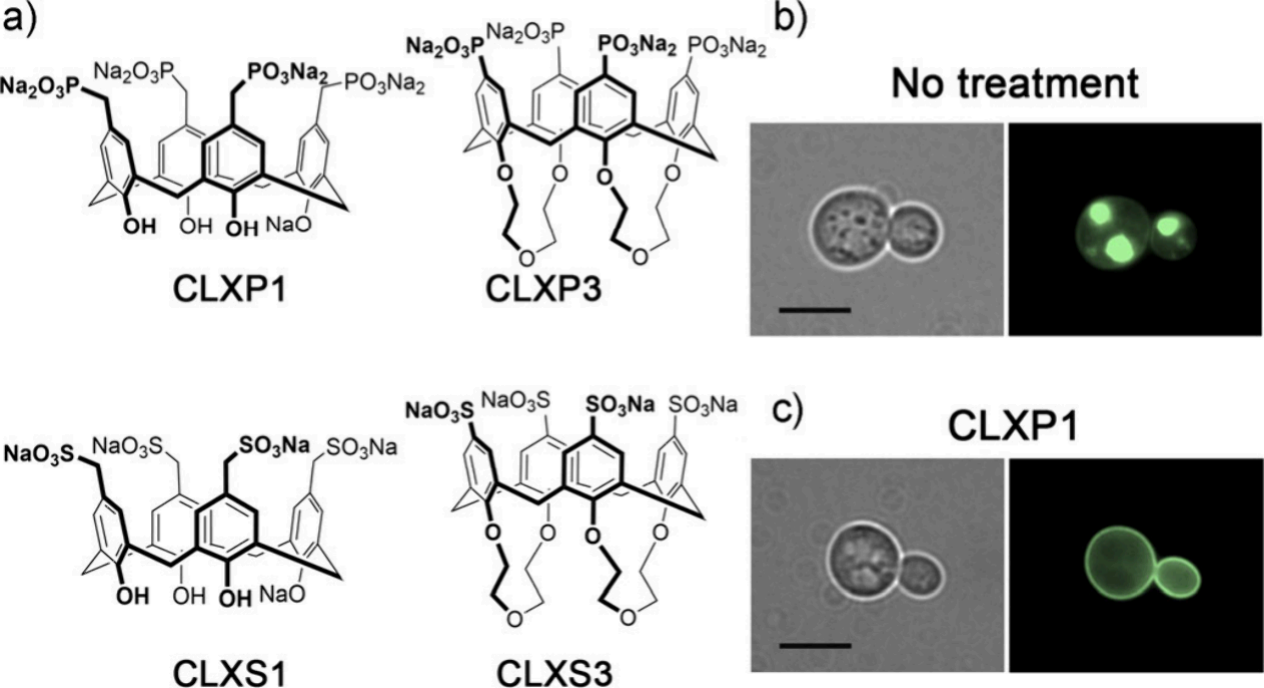
(a) Examples of water-soluble calixarenes used for selective disruption of α-synuclein aggregation. Microscopy analysis of cells overexpressing syn-GFP (HiTox strain) grown for 48 h at 28 °C in the (b) absence or (c) presence of **CLXP1**. Reproduced with permission from ref 6. Available under a CC-BY 4.0 license. Copyright 2026 Davide Dell’Accantera, Giulia Piccinini, Cristina Ciabini, Isabella C. Felli, Stefano Volpi, Nelson Marmiroli, Francesco Sansone, Roberta Ruotolo.

The combination of both phosphonylation at the upper rim and free hydroxyls at the lower rim provide the best synergy between aggregation disruption and antioxidant properties, showing the greatest cytoprotective effects.

A simple, dual-function calixarene is capable of recognition of α-synuclein in yeast cells with enough affinity and site-selectivity to limit aggregation and toxicity, while exploiting off-target mechanisms to reduce oxidative stress.

Overall, this manuscript elegantly shows the possible biomedical applications of synthetic receptors as therapeutics, and also illustrates the challenges. A simple, dual-function calixarene is capable of recognition of α-synuclein in yeast cells with enough affinity and site-selectivity to limit aggregation and toxicity, while exploiting off-target mechanisms to reduce oxidative stress. However, small changes in structure change the efficacy of the system significantly, and in unexpected ways—this system is specific for this particular purpose, and broadening the target scope or applying it *in vivo* will require significant effort to optimize. This is, however, an interesting and impactful application of simple synthetic receptors in a complex environment.
